# The Importance and Essentiality of Natural and Synthetic Chelators in Medicine: Increased Prospects for the Effective Treatment of Iron Overload and Iron Deficiency

**DOI:** 10.3390/ijms25094654

**Published:** 2024-04-25

**Authors:** George J. Kontoghiorghes

**Affiliations:** Postgraduate Research Institute of Science, Technology, Environment and Medicine, Limassol 3021, Cyprus; kontoghiorghes.g.j@pri.ac.cy; Tel.: +357-26272076

**Keywords:** chelation in medicine, iron overload, iron deficiency, natural chelators, chelating drugs, deferiprone, ferric iron tri-maltol

## Abstract

The supply and control of iron is essential for all cells and vital for many physiological processes. All functions and activities of iron are expressed in conjunction with iron-binding molecules. For example, natural chelators such as transferrin and chelator–iron complexes such as haem play major roles in iron metabolism and human physiology. Similarly, the mainstay treatments of the most common diseases of iron metabolism, namely iron deficiency anaemia and iron overload, involve many iron–chelator complexes and the iron-chelating drugs deferiprone (L1), deferoxamine (DF) and deferasirox. Endogenous chelators such as citric acid and glutathione and exogenous chelators such as ascorbic acid also play important roles in iron metabolism and iron homeostasis. Recent advances in the treatment of iron deficiency anaemia with effective iron complexes such as the ferric iron tri-maltol complex (feraccru or accrufer) and the effective treatment of transfusional iron overload using L1 and L1/DF combinations have decreased associated mortality and morbidity and also improved the quality of life of millions of patients. Many other chelating drugs such as ciclopirox, dexrazoxane and EDTA are used daily by millions of patients in other diseases. Similarly, many other drugs or their metabolites with iron-chelation capacity such as hydroxyurea, tetracyclines, anthracyclines and aspirin, as well as dietary molecules such as gallic acid, caffeic acid, quercetin, ellagic acid, maltol and many other phytochelators, are known to interact with iron and affect iron metabolism and related diseases. Different interactions are also observed in the presence of essential, xenobiotic, diagnostic and theranostic metal ions competing with iron. Clinical trials using L1 in Parkinson’s, Alzheimer’s and other neurodegenerative diseases, as well as HIV and other infections, cancer, diabetic nephropathy and anaemia of inflammation, highlight the importance of chelation therapy in many other clinical conditions. The proposed use of iron chelators for modulating ferroptosis signifies a new era in the design of new therapeutic chelation strategies in many other diseases. The introduction of artificial intelligence guidance for optimal chelation therapeutic outcomes in personalised medicine is expected to increase further the impact of chelation in medicine, as well as the survival and quality of life of millions of patients with iron metabolic disorders and also other diseases.

## 1. Introduction

The regular supply of essential nutrients including metal ions such as zinc, copper and iron is necessary for normal physiological functions and activity [1,2,3,4,5,6]. The maintenance of a specific range of concentration of these essential metal ions in tissues ensures healthy living. In contrast, deficiency or metabolic imbalance of such metals is associated in many cases with serious clinical conditions [6,7,8,9,10,11]. The metabolic imbalance of metals could be caused by the absence or malfunction of homoeostatic controls, genetic and metabolic abnormalities, irregular dietary supply and other causes [1,2,3,4,5,6,7,8,9,10,11].

Iron is not only required by all the cells of the body but also by symbiotic and exogenous microbes and all types of cancer cells. It is utilised for many functions and physiological processes, including energy transduction, and also for the transport, storage and utilisation of oxygen [1,2,3,10,11]. Under normal physiological conditions, iron balance is strictly controlled in humans and is maintained basically as a result of re-utilisation and equivalence between the rates of dietary absorption of iron and iron excretion or losses from the body.

The abnormalities associated with iron homeostasis and imbalance can lead to many different diseases including iron deficiency anaemia (IDA), which affects about a third to a quarter of the world’s population [10,11,12,13,14]; the anaemia of chronic disease (or anaemia of inflammation) [15,16,17,18,19]; other changes related to the metabolism, supply and distribution of iron to the haematopoietic tissues; organ function; in ageing; gastrectomy; as well as pharmacological and other interventions [20,21,22]. Furthermore, abnormalities of iron metabolism are observed in many genetic diseases including idiopathic haemochromatosis affecting one in ten persons of the Caucasian population [23,24,25,26,27], the haemoglobinopathies such as thalassaemia major (TM), which has the highest mortality and morbidity rate in comparison to any other form of metal intoxication worldwide [28,29,30,31], and also in haematopoietic stem cell transplantation widely used for many genetic disorders and haematological malignancies [32,33,34,35,36].

There are several toxicological aspects in relation to iron metabolism, where, for example, iron is considered as the major catalyst of free radical (FR) and reactive oxygen species (ROS) production in biological systems, which can cause biomolecular damage and progressively cellular damage as a result of toxicity related to oxidative stress [37,38,39,40]. The latter form of toxicity has also been associated with an increase in tissue damage in many pathological conditions including the initiation and progression of cancer [37,38,39,40,41,42,43,44,45,46,47].

One of the most recent major findings in relation to iron metabolism and iron-induced FR processes is the discovery in the last 12 years of ferroptosis [47], a new programmed cell death process, which is different from other processes involved in programmed cell death such as apoptosis and necrosis [47,48,49,50,51,52,53]. Ferroptotic programmed cell death is characterised by the induction of cell damage through iron-catalysed FR reactions leading to cell membrane lipid peroxidation, thus linking the associated metabolic pathways of iron metabolism and FR pathology [47,54,55,56,57]. In this context and over the last 12-year period, ferroptosis has been identified in almost all pathological conditions including all stages and types of cancer [47,58,59,60,61,62,63,64,65,66,67,68,69,70,71,72,73,74,75]. Different characteristics are observed in each disease where ferroptosis is implicated, including kidney, cardiac and neurodegenerative diseases, COVID-19 and many infections [76,77,78,79,80,81,82]. Most importantly, the inhibition of iron-induced oxidative stress toxicity damage by iron-chelating drugs and antioxidants has been considered as a major new strategy for the design of new drugs for the modulation of ferroptosis and the treatment of all associated diseases [48,83,84,85,86,87,88].

Chelators (Chele, greek χειλή—claw of a crab) play many other important roles in biology and in medicine [11]. Metal ions including iron are always found bound to ligands containing oxygen, nitrogen and sulphur in biological systems. These three electron donor atoms are involved in coordinating covalent bond formation with the metal ions. Most importantly, all metal-associated biological processes and activities are expressed and function through metal binding with different ligands. Chelators are organic molecules possessing two or more ligands, which have high affinity and can bind metal ions forming a chelator–metal complex composed of a ring with the metal ion as the closing member. The affinity of various ligands and the stability of the iron or other metal ion–chelator complexes are different but specific in each case. In biological systems, these differences lead to a continuous competition between metal ions for ligand and chelating binding sites [89,90]. Overall, both the ligands involved in metal binding and the metal complexes being formed play important roles in living systems.

Many more categories of molecules with chelating or metal binding properties are known, which can affect all processes involving metal ions. For example, many natural dietary molecules and drugs with iron-binding properties may have implications on iron absorption or excretion, as well as associated diseases including iron deficiency, iron overload and also iron-related metabolic processes such as ferroptosis [11,89,91]. Most importantly, iron-chelating drugs and iron–chelator complexes with specific properties, including those already regulatory approved and widely used in medicine, offer therapeutic solutions for many diseases associated with iron metabolic imbalance and toxicity [11,89,91].

In this review, recent advances related to the essential role of chelating drugs and chelator–iron complexes in the treatment of transfusional iron overload and iron deficiency anaemia, respectively, as well as their therapeutic implications on other common diseases of iron metabolism, are discussed in the biochemical, pharmacological and clinical context with major emphasis on the prospect of introducing improved and more effective therapies. Similarly, the implications of other factors affecting or modifying the therapeutic activity of chelating drugs and chelator–iron complexes, including improved drug combinations for designing optimal therapies and future strategies, are also discussed. This timely review approach in relation to the importance and essentiality of iron chelation is particularly relevant for rapidly expanding research fields, such as ferroptosis and associated diseases, and also many other diseases with no effective treatments such as cancer and neurodegenerative disorders.

## 2. Iron Metabolism and Iron Imbalance

Under normal physiological conditions, iron metabolic balance in humans is achieved and maintained due to the presence of specific metabolic pathways, proteins and transcription factors, which have been evolved for the uptake, distribution, utilisation, recycling and excretion of iron [1,2,3,10,11]. However, this balance can be modified or overturned because of different dietary, pharmacological, genetic, environmental and other factors, as well as other abnormalities such as different diseases, abnormal organ function, excessive bleeding, etc. Most of these changes require pharmacological intervention to restore iron balance. In this section, the basic iron properties and metabolic pathways responsible for iron balance, as well as major causes of iron imbalance in humans, will be introduced.

### 2.1. Iron Metabolism and Regulation of Iron in Humans

In physiological conditions, iron is always found bound to different ligands, mainly as ferric (Fe^3+^ or Fe (III)) or ferrous (Fe^2+^ or Fe (II)) iron forms [11]. Aqueous ferric iron is sparingly soluble at physiological pH, where it precipitates into ferric oxyhydroxide polymeric complexes with a high stability constant (log K = 38) and with only trace amounts of soluble ferric iron (10^−18^ mol/L) [11]. Aqueous ferrous iron is more soluble than ferric iron but is rapidly oxidised to ferric iron at physiological pH (unpublished observation). The solubility of iron increases at acidic pH and in the presence of iron-binding ligands and especially chelators [11].

Iron metabolism in humans is well regulated and controlled. It is also characterised by specific mechanisms, metabolic pathways and functional proteins, some of which have been evolved for the control of iron at various stages including its uptake from the gastrointestinal tract, distribution and utilisation in different organs and cells, as well as for its recycling and excretion [1,2,3,10,11].

In the context of cellular iron metabolism, different requirements of iron are needed and utilised by each cell, which depend on the fulfilment of specific biological functions and also the ability to store increased quantities of iron [1,2,3,10,11]. The transfer of iron in blood and its donation to all the cells of the body is accomplished by the iron transport protein transferrin, which has two chelating sites and can bind two iron (Fe^3+^) molecules, one in each site. The intracellular transfer of iron by transferrin is mediated through transferrin receptors, which are present on cell membranes [11,92,93,94,95,96]. Following endocytosis, iron is released at acidic pH from the transferrin/transferrin receptor complex from the endosome into an intracellular ‘low-molecular-weight (LMWt) iron pool’ composed from LMWt physiological natural chelators, for instance, citrate and cysteine, which are utilised in the cell for the turnover of iron containing proteins or transferred for iron storage [11,97]. Intracellular iron storage in all cells is accomplished by ferritin, a hollow protein sphere, one molecule of which can store up to 4500 molecules of ferric iron molecules in the form of ferric oxyhydroxide phosphate complexes [98,99,100,101,102]. Intracellular iron is also stored in haemosiderin, which is a cluster of ferritin molecules with a broken protein shell and exposed iron deposits [11,103]. In general, haemosiderin concentration predominates over ferritin in iron-loaded conditions [11,103,104,105,106]. Different iron storage capacities are observed in each organ with the liver, and to a lesser extent the spleen, predominating over other organs in iron storage levels, especially in chronically RBC-transfused patients [103,107,108,109].

In normal physiological conditions, iron is acquired by humans from different food constituents present in the gastrointestinal tract with the enterocyte playing a major role in the regulatory control of iron absorption. Several steps and metabolic pathways are involved in the uptake and utilisation of dietary iron. The general mechanism of dietary iron absorption is thought to involve initially the conversion of ferric iron forms to ferrous iron. This process takes place through the activity of a ferroreductase protein present at the cell surface of the enterocyte. The intracellular transport of iron in the enterocyte is accomplished by the apical divalent metal transported protein (DMT1) [1,2,3,10,110]. Once inside the enterocyte, iron is thought to be transferred into the LMWt iron pool and also into ferritin. The next step of the iron absorption pathway involves the transfer of the absorbed iron from the enterocyte into plasma, which is mostly regulated by the interaction of the protein ferroportin and the protein hormone hepcidin. Ferroportin, present at the basolateral membrane of the enterocyte, is responsible for the release of iron from the enterocyte into the circulation. The rate of release of iron into the circulation by ferroportin is subject to the regulatory control of hepcidin, which interacts with ferroportin. In particular, hepcidin can bind ferroportin, causing its internalisation and degradation within the enterocyte, thus preventing the release of iron into plasma [111,112,113,114,115,116]. Iron trapped in the enterocytes returns in the gut lumen following the shedding of the enterocytes, which occurs every few days. In contrast, the export of iron by ferroportin in plasma allows for its uptake by transferrin through mediation by LMWt chelators such as citrate and its transfer to all the cells of the body [1,2,3,11].

The iron regulatory protein hepcidin is produced in the liver and is responsible for other metabolic pathways, for example, the control of iron release into plasma from other cells such as macrophages [1,2,3,11,113,114,115]. The regulatory controlled mechanisms involving hepcidin could affect the overall rate of iron distribution in the body and most importantly the transfer of iron to the haemopoietic tissues for the production of haemoglobin. However, abnormalities in hepcidin function could lead to conditions of iron metabolic imbalance including iron overload, the anaemia of chronic disease and also IDA [1,2,3,111,112,113,114,115,116].

Iron absorption can also be affected by changes in transcription and other factors related to the expression DMT1 and ferroportin, which are expressed differently in various organs such as the duodenum, liver, haemopoietic tissues and kidneys [110,117,118,119]. Similarly, several other factors are also involved including the signal for increased intestinal iron absorption by regulatory molecules sensing the iron stores. Furthermore, other regulatory mechanisms appear to participate in the control of the production of haemoglobin in the haemopoietic tissues and for preventing anaemia. Overall, it appears that different regulatory molecules and transcription factors influence the uptake of iron from the gastrointestinal tract and its distribution to the haemopoietic and other tissues, playing a major role in iron balance [1,2,3,111,112,113,114,115,116,117,118,119].

### 2.2. Iron Distribution and Iron Balance

A continuous supply of iron is available from gastrointestinal absorption and re-utilisation from effete RBCs [3,10,11]. Most iron distributed in the human body (4.0 to 5.0 g) is mainly in haemoglobin (2.3–2.6 g) in RBCs in the blood and myoglobin (0.32–0.40 g) in muscle, in both of which iron is in the ferrous state (Fe II) in a complex form with a protoporphyrin ring (haem). Less iron in the polynuclear ferric (Fe III) oxyhydroxide phosphate complex form is found in the storage proteins ferritin (0.7 g) and haemosiderin (0.3 g); found mainly in the liver, spleen, muscle and bone marrow; and even smaller amounts in the proteins cytochromes (17 mg), catalase (5 mg), transferrin (4 mg) and non-haem iron-containing enzymes (0.1 g) [3,10,11].

Under normal physiological conditions, body iron balance is generally maintained through the endogenous iron turnover and re-utilisation [1,2,3,10,11,20,92,93,94,95,96,97,98] and when the rate of iron absorption from dietary sources is equivalent to the rate of iron excretion or loss [20,22,23,120]. It is estimated, for example, that about 2 mg (10–20%) of iron is absorbed from the gastrointestinal tract daily from a typical Western diet and the same amount is excreted or lost [20,121]. However, this balance is affected in many cases by increased iron requirements and utilisation for growth, e.g., in teenagers and pregnant women; in cases of blood loss, e.g., in blood donors; and in cases of iron loss, e.g., in long distance runners [122,123,124]. Body iron requirements may also depend on several other parameters including age, gender, lifestyle, sport activity, stage of health, etc. [10,11,124,125,126]. In particular, there are many different dietary iron variations and requirements between individuals and populations worldwide affecting iron balance [11,127,128,129]. For example, it is estimated that the daily iron requirements in adult men and post-menopausal women increase to about 8 mg, whereas in pregnant women they increase to about 27 mg, adult women to 18 mg, breastfeeding women to 9–18 mg and for teenage boys and girls to about 11 and 15 mg, respectively [121,124,130,131]. The insufficient supply of dietary iron in all the above cases of increased iron requirements could eventually lead to IDA, unless treatment with iron supplements becomes available.

The clinical manifestations of IDA include serious complications such as increased child and maternal mortality. Pregnancy and cardiac complications are also very common, and general symptoms include fatigue, reduced physical and mental performance, paleness, koilonychia, etc. [12,13,14,121]. The clinical symptoms of iron deficiency are mostly transient provided that treatment with iron becomes available through pharmaceutical iron supplements, which are widely available, and/or dietary changes, including meals containing sufficient amounts of absorbable iron dietary components.

In many cases mainly regarding IDA, the minor changes in iron balance could be easily restored, suggesting the presence of mechanisms of iron homeostasis and balance maintenance. This homeostatic control could be observed, for example, in blood donors or long distance runners, where the loss of iron is gradually restored without pharmaceutical intervention but from increased dietary iron absorption [122,123]. In contrast, imminent RBC transfusion is required following excessive bleeding, for example, as a result of surgery, war injuries or motor accidents, where the substantial loss of blood and iron cannot be rapidly restored. In contrast, in patients who receive excess iron, e.g., as result of a small number of RBC transfusions, iron balance is usually restored due to increases in iron excretion or loss [11].

In many cases, body iron imbalance and especially IDA could be established long term. For example, in the case of malnutrition and in vegetarian populations with IDA, the rate of iron losses may be greater than the rate of iron absorption due to the insufficient or ineffective intake of dietary iron. In contrast, the reverse is taking place, for example, in Bantu siderosis, where the rate of iron absorption is greater than the rate of iron losses. In individuals suffering from Bantu siderosis, excess iron is absorbed from iron utensils which are used for cooking [11,132,133,134,135].

It appears from the above observations that iron balance is affected by the overall rate of iron absorption and body iron intake, as a result of the quantity and quality of iron entering the gastrointestinal tract. Similarly, several other factors and dietary habits, such as different food types and drugs as well as the level of water and alcohol intake, could also influence the iron absorption process. Most importantly, it also appears that the presence of low dietary iron in vegetarian meals can overall cause a reduction in the intake of iron in vegetarian populations. In contrast, sufficient amounts of iron are absorbed because of the presence of high concentrations of haem in meat-eating populations, which is better or more readily absorbed from the gastrointestinal tract in comparison to other dietary iron forms [127,128,129].

### 2.3. Genetic and Other Diseases of Iron Imbalance and Distribution

Abnormalities in iron metabolism and body iron distribution are observed in many genetic and other diseases affecting millions of humans worldwide. In addition to IDA and the other diseases mentioned above, there are many genetic diseases with abnormal body iron intake and distribution. One such genetic disease is idiopathic haemochromatosis, which is characterised by increased dietary iron absorption leading gradually, over many years, to iron overload with iron toxicity manifested towards the middle age or late stages of life [23,24,25,26,27]. The toxic side effects of iron overload in idiopathic haemochromatosis include liver damage, arthritis, cardiovascular damage, diabetes and in some cases hepatocellular carcinoma [136,137,138,139,140]. Idiopathic haemochromatosis affects one in ten persons of the Caucasian population and the toxic side effects of excess iron overload can be prevented if the disease is detected early in life and the patients are treated by regular blood removal phlebotomy programmes [11,23,24,25,26,27].

Excess iron absorption from the gastrointestinal tract and the cause of body iron overload are also observed in thalassaemia intermedia (TI) of alpha, beta and other haemoglobin subtype chain variants, as well as other haemoglobinopathies with refractory anaemia due to ineffective erythropoiesis and insufficient production of normal RBCs [141,142,143,144]. In this case, increased iron absorption is driven from the increased erythropoietic activity of the bone marrow to compensate for the anaemia. The excess iron absorbed is deposited primarily in the liver and the spleen and also other organs. A periodic increase in iron absorption is also observed in TM, especially in the period prior to RBC transfusion due to ineffective erythropoiesis and inadequacy to compensate for the anaemia [141,142,143,144].

There are many other categories of anaemic patients with different pathophysiology in addition to those described above. In particular, a major category with millions of patients worldwide are those suffering from the ‘anaemia of inflammation’ or otherwise the anaemia of chronic disease. This category includes patients with inflammatory, neoplasmic and infectious diseases. Although sufficient iron is absorbed in the body in most of the different categories of patients with the anaemia of chronic disease, eventually, a lot of iron is diverted to the reticuloendothelial system, from where it cannot become readily available to the haemopoietic tissues for the production of haemoglobin [15,18,19].

Another major category of patients with iron overload are those receiving chronic RBC transfusions due to refractory anaemia. In this category, excess iron from effete RBCs is deposited in all major organs causing iron overload toxicity damage [145]. This group includes in addition to haemoglobinopathies different patient categories such as haematopoietic stem cell transplantation, many haematological and other malignancies and genetic disorders [28,29,30,31,32,33,34,35,36]. In particular, in relation to haemoglobinopathies, most TM patients have excess iron deposition and associated iron toxicity damage in different organs including the liver, spleen, heart, pancreas, thyroid, pituitary gland, gonads and joints [146]. As a result of excess iron toxicity, TM is considered to be the most well-known disease related to iron or general metal intoxication with the highest mortality and morbidity rate in the world [145,146].

There are many other categories of patients affected by iron imbalance such as those with haemolytic diseases, kidney or other organ damage or where iron is diverted from the haemopoietic tissues as a result of, for example, infection, malignancy or inflammation causing anaemia. In most of such cases, insufficient iron is available for the production of haemoglobin [147,148,149]. Other causes of iron imbalance include abnormalities in the structure, function and rate of production of proteins related to iron metabolism such as transferrin, ferroportin, hepcidin, DMT1 and erythropoietin. Similarly, abnormalities in iron metabolism and iron balance could be caused by different natural products and drugs.

A major role in iron metabolism and the treatment of iron overload and also IDA is played by iron-chelating drugs and other chelators. In this context, iron chelators could be used in many cases for modifying iron’s metabolic effects and related diseases. In particular, the increased interest in chelators observed following the discovery of ferroptosis highlights the importance of iron metabolism in normal cell survival and the prospect of iron chelation therapy interventions in many other diseases in addition to iron overload and iron deficiency.

## 3. Iron Chelation, Natural Chelators and Chelating Drugs

**Figure 1:**
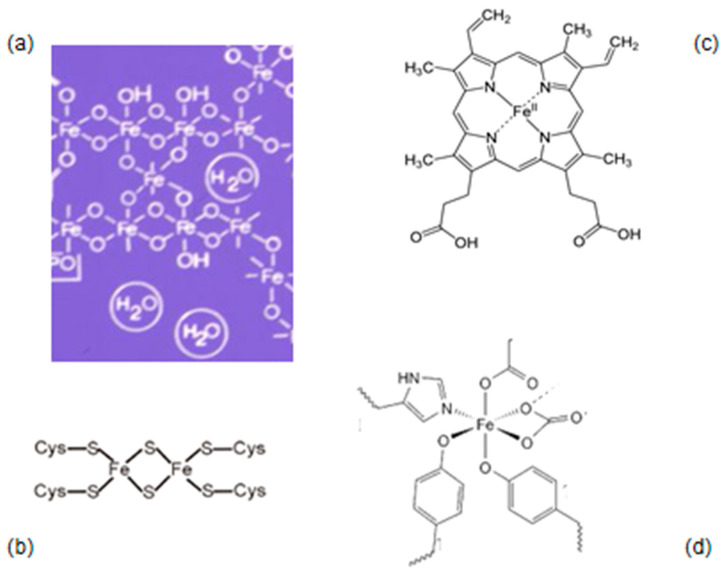
The chemical composition of iron complexes in different proteins. In ferritin, iron (Fe III) is deposited as a polynuclear oxohydroxy phosphate iron complex (**a**). In the iron containing protein xanthine oxidase, iron is present as an iron–sulphur complex involving two molecules of iron (Fe III and Fe II) and six of sulphur with four sulphur molecules derived from cysteine (cys) (**b**). In haemoglobin, the haem complex involves four nitrogen molecules from a protoporphyrin ring and ferrous iron (Fe II) in the centre (**c**). In transferrin, iron (Fe III) in the iron-binding site is bound by five oxygen molecules from two tyrosines, aspartic acid and carbonate, and also a nitrogen molecule from histidine (**d**). (Some of the structures of the iron complexes in the figure have been copied from reference [11]).

In biological systems, iron is always found bound to adjacent ligands such as those containing =O, –OH, –N and –SH, which possess electron-donating atoms such as oxygen, nitrogen and sulphur, ensuring diversity in activity and function. In particular, the same electron-donating atoms are present in transferrin which mostly contains oxygen-based ligands; haemoglobin and haem enzymes containing nitrogen-based ligands; and aconitase containing sulphur-based ligands (Figure 1) [11,89,150,151,152,153]. In addition, many dietary molecules and chelating and other drugs possess similar ligands with different chelating capabilities for binding iron [11,89].

Structural and biochemical function changes of iron complexes in vivo, including those of iron-containing proteins, can be caused as a result of different factors and interactions. On the molecular level, such changes may take place from interactions with other chelators, ligands, metal ions, etc. Similar changes in the structure of iron complexes including iron-containing proteins can affect different processes associated with iron metabolic pathways [11,89].

### 3.1. Natural Biomolecules with Iron-Chelating Potential

There are many biomolecules involved in the binding, solubility and carriage of iron in biological fluids and in cells. Most importantly, the major role of iron uptake and transfer in plasma is played by the chelating protein transferrin which ensures the rapid and secured supply of iron to all cells (Figure 1). Iron uptake by transferrin is mostly accomplished by LMWt donor chelating molecules such as citrate in plasma [11,89,154,155]. Similarly, iron binding and complex formation by LMWt chelators occur intracellularly, which are components of the ‘LMWt iron pool’, including amino acids, nucleosides, carbohydrates, phosphates, ATP, glutathione, etc., all of which provide sufficient amounts of iron in a soluble form to be used for incorporation in iron-containing proteins or for iron storage in ferritin [11,89,97].

Iron mobilisation by transferrin in plasma also has many other roles including antimicrobial and antioxidant effects, since iron is required for microbial growth and free radical formation, oxidative stress and ferroptosis [92,93,94,156,157,158]. These effects are also observed with lactoferrin, the sister iron-chelating protein of transferrin found in secretions such as milk, tears, vaginal and other fluids and also in neutrophils [159,160,161,162].

Ascorbic acid is another example of a natural chelator with many and different interactions with iron, iron metabolism and pharmaceutical applications [163,164,165,166]. Ascorbic acid is a potent antioxidant and reducing agent, affecting iron absorption and also iron excretion [167,168,169,170]. For example, it can increase iron absorption during co-administration with iron and also is a component of pharmaceutical formulations such as ferrous ascorbate [171,172,173]. In contrast, it can be used in combination with the iron-chelating drug DF for enhancing iron excretion [174,175]. Chelating, redox and other effects on different aspects of iron metabolism including iron absorption and excretion have also been reported by many other naturally occurring phytochelators similar to ascorbic acid such as different polyphenols [176,177,178,179,180].

Another important group of naturally occurring chelators affecting iron metabolism is the microbial siderophores, which are produced by microbes for the acquisition of iron from the surrounding environment, which is essential for their survival and proliferation. The interaction of microbial siderophores with iron could influence transferrin iron transport and other iron metabolic pathways, including iron absorption and excretion, as well as the proliferation of microbes [181,182,183].

Iron binding by chelators in biological systems is a complex process influenced by many factors such as competition by other chelators or metal ions and also different metabolic processes and conditions present in each cell type or tissue. In this context, metal complexation reactions provide important information on basic parameters related to the affinity of natural and other chelators for iron and the stability of the iron complexes under different conditions, including the acidic environment of the stomach, competition with other metals and transferrin and different interactions with other proteins.

### 3.2. Properties and Effects of Natural Chelator–Iron Complexes

There are many types of iron complexes which are present and also formed in the human body including many iron containing proteins, LMWt chelator complexes and also iron complexes with dietary chelating molecules such as ascorbic acid and other phytochelators. Similarly, there are many drugs with iron-chelating capacity and also iron-containing drugs which are widely used for the treatment of IDA [11]. All these iron complexes have different physicochemical, metabolic and other characteristics, which are involved in different interactions with other biological components including metal ions and other natural chelators (Table 1).

Natural and synthetic chelators form complexes that are stable in plasma and other biological fluids, where in some cases they can exchange their iron with other chelators (trans-chelation) including transferrin, e.g., the citrate–iron complex donates its iron to transferrin; others may not exchange their iron, e.g., the DF iron complex does not donate iron to transferrin; or they partly exchange and form mixed complexes, e.g., maltol iron–transferrin complex [123,151,157]. In general, the stability, interactions and other properties of chelator–iron complexes are governed by thermodynamic and kinetic parameters, which could affect physiological processes and have different implications in health and disease [151,152,153].

The functional and other properties of each of the iron complexes are unique. For example, haem, which is an endogenous lipophilic protoporphyrin ring iron complex, plays important roles as a functional part in haemoglobin for oxygen transport and many other haem-containing proteins for energy transduction, and it also has other functions (Figure 1, Table 1) [11]. Haemoglobin is essential for respiration and carries oxygen when iron is in the ferrous form, whereas methaemoglobin where iron is in the ferric form is unable to bind and carry oxygen. Following the catabolism of haemoglobin and other haem iron-containing proteins, the haem ring is eventually cleaved intracellularly by haem oxygenase (HO-1) producing ferrous iron, carbon monoxide and biliverdin, with the latter subsequently being reduced to bilirubin [183,184,185,186]. The iron released from the breakdown of haem is mostly incorporated in ferritin and also the LMWt iron pool to be utilised for different functions. The increased breakdown of haemoglobin and excessive release of bilirubin, which has a yellow colour, is characteristic of jaundice [187].

Another important property of haem is in relation to iron absorption. In this context, haem that is found mainly in meat products enhances iron absorption, since in general the haem iron complex is more lipophilic and readily absorbed in comparison to other less lipophilic dietary iron complexes of different natural chelators [187,188,189,190]. It is important to note that in contrast to haem, nitroso haem found in processed meat is a known carcinogen implicated in colorectal and other cancers [191]. The release of haem into the blood stream is also toxic and the toxicity is alleviated by the specific plasma protein haemopexin, which is expressed in the liver, has high affinity to haem binding and can remove haem from plasma [192]. Similarly, haptoglobin is another plasma protein which binds and removes free haemoglobin in blood [193]. In both cases, heam iron is released from the metabolism of these protein complexes and ferrous iron is mostly incorporated in ferritin [11,184,187].

Many other lipophilic chelators forming lipophilic iron complexes including the natural plant products 8-hydroxyquinoline, omadine and maltol have been shown to increase iron absorption in vivo and in clinical studies [11,124,194,195]. Similarly, the iron-chelating drugs DF, deferiprone (L1) and deferasirox (DFRA) also affect iron absorption, with the hydrophilic DF and L1 both inhibiting this process, whereas the lipophilic DFRA promotes the absorption of iron and other metals [11,194]. In particular, oral DF is widely used as a first-line drug for inhibiting the absorption of iron in cases of iron poisoning [196,197].

In general, iron complexes including those with natural food components and also drugs possessing iron-chelation capacity can affect the general interactions and also the transfer properties of iron across different cells including those of the gastrointestinal tract and also other parts of the body (Table 1) [11,20,21,91,198,199,200]. Similarly, different pharmacological, toxicological and therapeutic characteristics are observed between iron complexes, which depend on various parameters, including the size, solubility, lipophilicity and the stability of the complex, as well as other physicochemical parameters [11,150,151,152,153]. In particular, the metabolism of most iron and other metal complexes is influenced by the presence of the iron-chelating proteins lactoferrin and transferrin. Similarly, different endogenous LMWt chelating molecules and competing metal ions such as zinc, copper and aluminium can also affect the stability of iron complexes and their metabolic characteristics [90,144,157].

Trans-metallation and trans-chelation may occur during the interactions and exchanges of other metals and other chelators with the chelator–iron complexes. In such cases, the displaced iron molecules enter the physiological iron metabolic pathways. Similarly, the chelator dissociated from the iron complex also follows different metabolic, pharmacological and toxicological pathways with specific metabolic routes, which are different in comparison to those observed in the case of a chelator–iron complex [123].

### 3.3. The Properties and Effects of Iron-Chelating Drugs

The primary role of iron-chelating drugs in medicine is the elimination of excess iron and associated toxicity arising mainly from chronic RBC transfusions and/or increased iron absorption, which have been implicated for the high morbidity and mortality observed in affected iron loaded categories of patients [29,31,32,33,34]. In this context, the ultimate aim of iron chelation therapy is the complete removal of excess toxic iron and the maintenance of normal physiological iron levels in iron-loaded patients [201].

**Figure 2:**
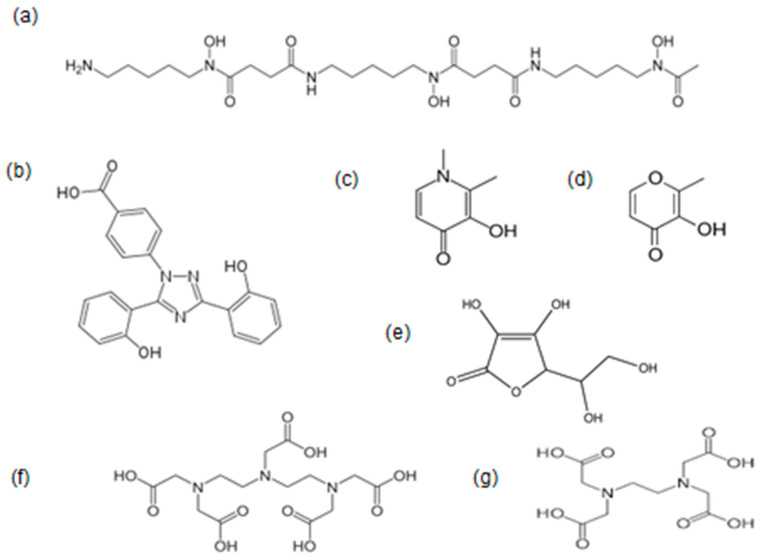
The chemical structure of the iron-chelating drugs and of some other chelators in clinical use. Deferoxamine is administered subcutaneously (**a**), deferasirox orally (**b**) and deferiprone orally (**c**). All three (**a**–**c**) are widely used for the treatment of iron overload in thalassaemia and also other similar conditions of transfusional iron overload. Maltol (**d**) and ascorbate (**e**) are natural phytochelators consumed by humans and used in the pharmaceutical industry. Diethylenetriaminepentacetic acid or DTPA (**f**) is used for plutonium decontamination in the nuclear industry and ethylenediaminetetracetic acid or EDTA (**g**) for general metal detoxification and in alternative medicine.

The regulatory approved drugs for the treatment of iron overload used worldwide are DF, L1 and DFRA (Figure 2). Their efficacy, toxicity and other properties have been previously reviewed [144]. In many cases, different combinations of these three drugs are also used as a method for enhancing the efficacy and reducing the toxicity of iron chelation therapy in comparison to chelating drug monotherapy [202,203,204].

Many other drugs with chelating ligands and potential for iron binding also influence iron metabolic pathways. Despite the fact that these drugs are not as effective as DF, L1 and DFRA in increasing iron excretion in RBC-transfused patients, their interactions with iron have other implications on iron metabolism and iron balance [205]. Examples of such interactions include those involving tetracyclines, where iron binding affects the absorption of both the drug and iron [206,207,208], and also aspirin, where its daily administration long term can cause IDA in the elderly [209]. Many similar drugs can also influence iron metabolism and toxicity and are used therapeutically in other diseases. In particular, the pro-drug dexrazoxane, widely used for reducing the cardiotoxicity of the anticancer drug doxorubicin, forms an EDTA-like iron chelator metabolite, which binds iron in the heart, implicated in doxorubicin toxicity [210].

In contrast to the chelating drugs DF, L1 and DFRA, the primary role of iron complexes with chelating drugs and other iron complex drug formulations is the increase in iron absorption and its utilisation for haemoglobin synthesis in the treatment of IDA (Table 1) [123,194]. In this case, the sufficient supply and intake of iron from iron complex formulations will be needed to maintain body iron balance and haemoglobin levels. Iron supplementation is widely available worldwide [211,212]. Most iron supplements for the treatment of iron deficiency contain formulations of oral ferrous or ferric iron and a sugar molecule such as gluconate, fumarate, maltose, sucrose, etc. Iron dextran (polymer of glucose) is also widely used for iv administration. Non-sugar formulations include inorganic ferrous sulphate and also ferrous ascorbate (Table 1) [123]. Most iron formulations are orally administered in a capsule, tablet or extended-release tablet or capsule, whereas few other formulations are administered as liquid preparations. Usually, the iron formulations contain about 30–100 mg elemental iron, most of which is not absorbed [123,194].

Despite the fact that the availability of iron from the above formulations is small in comparison to the administered amount of iron, it is in most cases sufficient for treating iron deficiency and bringing patients to iron balance and normal haemoglobin levels within a period of few weeks or months. In many cases, the non-absorbed iron from oral formulations can cause gastrointestinal toxicity, which in some cases cannot be tolerated and the treatment is discontinued [123,213,214]. Usually, an alternative oral iron formulation can replace the one causing the gastrointestinal irritation, whereas in extreme cases, injectable iron formulations or even RBC transfusions could be used to treat IDA.

## 4. Recent Advances in Chelator–Iron Complexes for the Treatment of Iron Deficiency Anaemia

Despite the fact that in the majority of IDA cases the treatment with existing iron formulations is satisfactory, there is a major scope for further improvements, especially in many non-responding categories of IDA patients. In such cases, more efficient iron formulations could be administered for increasing iron absorption and distribution in the haemopoietic tissues with reduced or no toxicity [123,213,214]. In this context, a large variety and selection of new oral and other iron formulations for the treatment of IDA are advertised very often in the mass media by the pharmaceutical and nutraceutical industries, usually claiming improved response in IDA patients. This commercial activity highlights the increasing demand and interest by a large number of IDA patients worldwide. However, there is no general consensus worldwide among physicians and there is also sometimes confusion among IDA patients for the selection and optimal use of the available iron formulations. In many cases, iron formulations are chosen by patients based, for example, on commercial promotion campaigns or in other cases the cheapest available option because of budget constraints, e.g., the use of inexpensive ferrous sulphate formulations, especially in developing countries (Table 1) [215].

A new scientific approach related to the design and development of improved iron formulations with higher efficiency and lower toxicity is the use of more specific, lipophilic chelator–iron complexes for all the different categories of IDA patients. This approach was proposed forty years ago but only recently obtained regulatory approval in the case of the ferric iron tri-maltol complex (feraccru or accrufer) [124,151,216]. The design, background route of development including in vitro, in vivo and clinical studies, as well as the mode of action and advantages in the treatment of IDA patients using the ferric iron tri-maltol complex formulation have been recently reviewed (Table 1) [124].

The efficacy and low toxicity of the ferric iron tri-maltol complex has been previously studied and compared with ferrous sulphate and other iron formulations in many categories of IDA patients including inflammatory bowel disease and pulmonary hypertension. Usually, treatment with the ferric iron tri-maltol complex involves the administration twice daily, before breakfast and the evening meal, of 30 mg of the iron maltol complex for up to 3 months. In such cases, significant increases in mean haemoglobin (e.g., from 106 to 126 g/L and from 107 to 136 g/L), in serum ferritin (e.g., from 13.1 to 33.6 μg/L and from 8.1 to 17.4 μg/L) and in transferrin saturation (from 7.5% to 31.5%) have been observed in different categories of iron-deficient patients involved in separate clinical studies [217,218,219,220,221,222,223]. In almost all the clinical studies, the ferric iron tri-maltol complex appears to cause both more rapid and high total level of iron absorption equivalent or greater to that seen with ferrous sulphate and also with an efficacy equivalent to that of intravenous iron formulations [224,225].

The mode of action of the ferric iron tri-maltol complex and other lipophilic iron–chelator complexes appears to be different from that of other iron formulations. It has been shown, for example, that iron transfer and donation, as well as increased haemoglobin production in erythroid cells by the ferric iron tri-maltol complex and other lipophilic ferric iron–chelator complexes, could proceed in the absence of transferrin and is independent of transferrin iron delivery [226]. Most importantly, clinical trials in different categories of IDA patients using the ferric iron tri-maltol complex have indicated better specificity in iron delivery and increased haemoglobin production in comparison to other ferrous or ferric iron formulations [217,218,219,220,221,222,223,224,225]. Furthermore, in contrast to other iron formulations, no serious toxicity related to the ferric iron tri-maltol complex has been reported in the clinical trials or the post-marketing surveillance of the pharmaceutical formulation so far [226,227,228,229,230,231].

The overall effects of the ferric iron tri-maltol complex and similar chelator–iron complexes on iron absorption depend mainly on the quantity of iron present in the formulation, as well as many other factors such as individual variations on absorption, distribution, metabolism, excretion and toxicity (ADMET) and also pharmacogenomic, proteogenomic, redoxomic and metallomic characteristics [232]. Furthermore, the absorption of iron from the ferric iron tri-maltol complex and other chelator–iron complexes is partly affected by the presence of other competing metal ions and also competing dietary molecules and drugs with iron-binding capacity [90,205,206,207,208,209,210]. Many other factors and conditions can also affect the level of iron absorption and iron distribution in individuals from oral iron formulations. These include changes in the normal function of the gastrointestinal tract, haematopoietic and other organs, as well as different diseases, ageing, nutrition, etc.

In addition to concerns about the toxicity of iron and its complexes, the chelating and other molecules involved in the iron formulations are also important components regarding the overall toxicity of the drugs used for the treatment of IDA. In this context, maltol released from the ferric iron tri-maltol complex is considered a safe component at the concentrations used, especially since it is a natural plant product widely used in the food industry and consumed by humans for more than sixty years (Figure 2) [233,234]. Similarly, the glucuronide conjugate of maltol, which is formed from the metabolism of maltol, is also considered a safe metabolic product [235].

The wide clinical and veterinary use of the gallium tri-maltol complex is an additional application of maltol metal complexes, which supports the general safe use of maltol in medicine [236,237]. Further clinical and other studies using maltol and other lipophilic chelator complexes for the specific targeting treatment of different IDA categories of patients are required, including the improved risk/benefit assessment for reducing the overall iron toxicity of different iron formulations.

## 5. Recent Advances in Iron-Chelation Protocols for the Complete Treatment of Iron Overload

The historical, pharmacological, toxicological and other aspects of the three chelating drugs DF, L1 and DFRA have been previously reviewed (Figure 2) [144,238]. The recommended ranges of doses of these drugs by the manufacturers in iron-loaded TM patients are 40–60 mg/kg/day for subcutaneous (sc) DF, 75–100 mg/kg/day for oral L1 and 20–40 mg/kg/day for oral DFRA [144]. There is no consensus on the use of the three chelating drugs and at present, different drug protocols, drug doses, drug formulations and drug combinations are used in different countries and clinics [232,238]. Furthermore, all three chelating drugs have differences in efficacy, toxicity profile, tolerance, site of action, the risk/benefit assessment and also cost. All these differences affect the overall mortality and morbidity rate of iron-overloaded patients worldwide [133,144].

The high rates of morbidity and mortality in TM and other categories of regularly transfused RBC patients are caused by iron overload toxicity and associated damage to the heart, liver, endocrine system organs and other organs [145,146]. In many developing countries, RBC-transfused TM patients not receiving chelation therapy die by the age of 20 years mostly from congestive cardiac failure caused by cardiac iron overload toxicity [29,30,31,239,240]. Epidemiological data in the UK have shown that mean survival in TM increased to 35 years following the introduction of sc DF, with congestive cardiac failure being the main cause of mortality [241]. The use of effective chelation therapy protocols can prevent or minimise iron overload toxicity and can substantially decrease the associated morbidity and mortality rate in TM and other RBC-transfused categories of patients [242,243,244]. In this context, the ultimate aim of iron chelation therapy in regularly transfused RBC patients is the removal of all excess toxic iron and the maintenance of normal iron store levels, which is characterised by the normal physiological range of serum ferritin (350 μg/L>), cardiac T2* (>20 ms) and liver T2* (>6.3. ms) MRI relaxation time levels [201].

**Figure 3:**
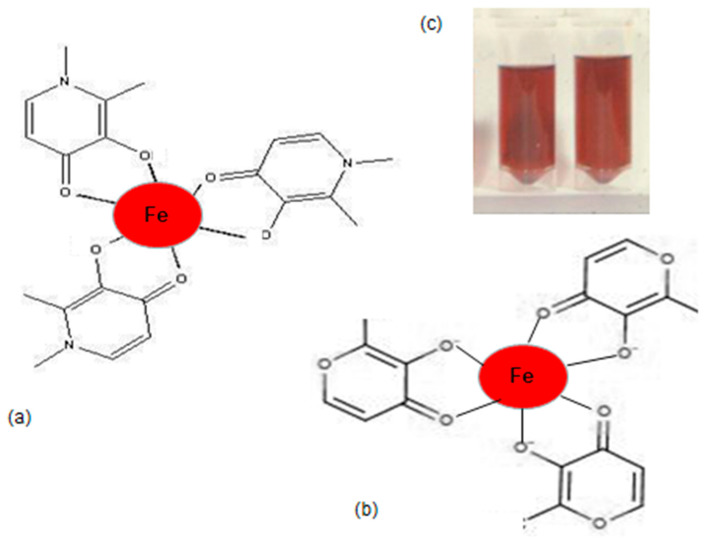
Iron complexes of deferiprone and maltol. Both deferiprone and maltol form octahedral 3:1 chelator–iron complexes at physiological pH. The ferric iron (Fe III) tri-deferiprone complex (**a**) has a characteristic orange-red colour as shown in the urine sample (**c**) of a thalassaemia patient treated with deferiprone. Similarly, the same colour is observed in the ferric iron tri-maltol complex at physiological pH (**b**).

Personalised medicine chelation protocols can ideally be selected for each patient based not only on general iron load parameters but also parameters such as drug efficacy and metabolism, toxicity aspects, compliance and also organ targeting [144,146,245]. However, there is bias and controversy in many cases regarding the selection criteria and use of chelating drugs for optimal therapy worldwide, which go beyond iron overload [232]. In addition, there is no consensus in the risk/benefit assessment and evaluation criteria for the use of each of the drugs. In particular, the cost of the drugs in the developing countries where most TM patients live is the main issue affecting chelation therapy [245,246]. Other parameters are also important among the chelating drugs, such as differences in the pharmacokinetic and metabolic profile. In this context, following administration, DF is rapidly cleared from blood in minutes, whereas L1 is cleared in about 6 h and DFRA in more than 19 h. Furthermore, there are differences in the excretion route of the chelator–iron complexes, with DFRA causing an increase in iron excretion almost exclusively in the faeces, whereas L1 is excreted exclusively in the urine and DF mostly in the urine and also some in the faeces (Figure 3) [144,155].

The use of effective chelation therapy protocols in iron-loaded TM patients and especially those involving L1 and L1/DF combination, as well as sometimes combinations including DFRA, appear in the last three decades to have caused a negative iron balance, lower morbidity and mortality, and life expectancy at levels approaching those of the general population in many cases [232,242,243,244,247,248]. In most cases, specific personalised chelation therapy protocols are designed and applied [232]. In particular, the choices of chelation protocols can range from an intensive administration, such as combinations of DF (40–60 mg/kg/day) and L1 (75–100 mg/kg/day) for heavily RBC-transfused iron-loaded patients, to cases where much lower doses and a reduced frequency of administration are used, including intermittent withdrawal of chelation, for example, in TM and TI patients who have achieved normal iron store levels [232]. In such cases, the continuation of high doses of chelatiing drugs can cause iron deficiency and also other clinical complications [249,250].

The prospect of designing personalised medicine chelation protocols in TM and other similar iron metabolic disorders began following the introduction of magnetic resonance imaging relaxation time T2* (MRI T2*) monitoring, which could identify the level of the iron load in many different organs [108,241,251]. The combination of the serum ferritin and MRI T2* for the estimation of iron load in different organs was instrumental in the introduction of successful personalised monitoring parameters for the efficacy of different chelation protocols and chelating drugs. In particular, this approach was used for the introduction of the International Committee on Chelation (ICOC) protocols of the L1/DF combination or L1, which appear to cause the complete elimination of iron overload from the heart, liver and spleen of TM patients with different levels of iron load in these organs [252,253,254,255].

In most TM patients, negative iron balance and complete clearance of excess iron in both the heart and the liver can be achieved in between 0.5 and 1.5 years using the ICOC protocol of oral L1 (75–100 mg/kg/day) and sc DF (40–60 mg/kg/day at least 3 days per week) [252,255]. The rate of iron clearance is faster when using higher overall doses of L1 and DF and in patients who are less heavily iron-loaded [252,253,254]. More intensive chelation in heavily iron-loaded patients can be achieved using intravenous DF in combination with L1. In contrast, lower overall doses and mostly L1 monotherapy are used in most TM, TI and other categories following the normalisation of the iron stores [253]. Excess iron removal from the liver but at a much slower rate from the heart appears to be effective usually at high doses (30–40 mg/kg/day) of DFRA [203,256,257]; however, this is not equivalent to that caused by DF, L1 and their combination [252,253,254]. Higher efficacy is observed when DFRA is used in combination with DF and L1 (Figure 2) [258,259,260].

The regular monitoring of iron overload toxicity and prophylactic measures for chelating drug toxicity are essential for the prognosis and survival of TM and other similar categories of patients [146,147,148,248,250]. In this context, the treatment of young TM and other iron-loaded categories of patients with serum ferritin lower than 500 μg/L is restricted for DFRA and DF due to toxicity implications [250].

Toxic side effects reported in iron-loaded patients treated with DFRA include renal, liver and bone marrow failure including agranulocytosis, as well as renal toxicity, skin rashes, gastric intolerance, etc. [261,262,263,264]. Increased morbidity and mortality have been reported in patients with non-iron-loaded conditions treated with DFRA [250,263]. In particular, the regular monitoring of kidney function in patients treated with DFRA is recommended and in some cases drug withdrawal is necessary especially for patients with persistent increases in serum creatinine levels [250,264].

Toxicity in non-heavily iron loaded patients has also been observed during treatment with DF. In this context, ocular and auditory toxicity in non-heavily iron-loaded TM or other categories of patients, as well as fatal mucormycosis in renal dialysis patients with normal iron stores, have been reported during treatment with DF [265,266,267]. Many other toxic side effects such as anaphylactic reactions, yersiniasis, local irritation and general non-compliance from the sc infusion of DF are also observed in iron-loaded TM patients [268,269,270].

The relative safety of L1 in comparison to DF and DFRA in TM, TI and other categories of iron-loaded patients with low or normal iron stores has been shown in thousands of patients in the last 30 years and also in studies in TM patients with normal iron stores exceeding 100 patient years [254,271]. In the last few years, the clinical studies using L1 in non-iron overloading diseases has been expanded to include neurodegenerative, cardiovascular, renal and infectious diseases, and also other categories of patients [272,273,274,275,276,277,278,279,280]. For example, during the clinical studies in Friedreich ataxia and non-diabetic glomerular disease patients, L1 was used at doses of 50–75 mg/kg/day for 6–9 months with very encouraging results and no serious toxic side effects [272,277]. However, the results from the use of much lower doses (2 × 10 mg/kg/day) in Parkinson’s disease patients were disappointing [281]. In relation to safety and toxicity regarding L1, the most serious toxic side effects reported in the last 30 years include neutropenia and reversible agranulocytosis affecting less than 5% and 1% of patients, respectively [144,146,271,282]. In this context, it is recommended that patients treated with L1 should be monitored weekly or fortnightly using a mandatory blood count for prophylaxis against these toxic side effects. Similarly, several other less serious toxic side effects of L1 have been reported including joint and musculoskeletal pains, gastric intolerance and also zinc deficiency. Zinc supplements are widely used in the latter case for prophylaxis of TM patients on a long-term treatment with L1 [144,146,282].

Overall, toxicity vigilance and prophylactic measures are very important parameters for ensuring the safety, therapeutic outcome and long-term survival of TM, TI, sickle cell disease and many other of iron-loaded and non-iron-loaded categories of patients treated with chelation therapy using the three different iron-chelating drugs.

Precision personalised medicine chelation treatments based on individualised, effective and safe chelation therapy protocols need to be designed and applied for treating the various categories of iron-loaded patients and also of non-iron-loaded categories of patients [146,252]. In both categories of patients, the continuous monitoring and adjustment of the iron-chelation protocols including monotherapies or combination therapies will be needed for optimal results and also until the therapeutic targets are achieved with minimal or no toxicity.

## 6. Future Strategies in the Treatment of Iron Deficiency Anaemia and Iron Overload Diseases

New developments in the areas of iron metabolism, iron chelation therapy and related diagnostic techniques could provide important information for the design of new improved therapeutic strategies for more effective treatments in iron overload, IDA and other diseases of iron imbalance. These therapeutic strategies could be designed based on further information from in vitro, in vivo and clinical studies of the interactions of different chelators with all the sites and stages of iron metabolism including molecular iron, iron-containing proteins and other proteins involved in iron metabolic pathways.

A major area requiring further investigation is in relation to the mode of action and other properties of endogenous and exogenous chelating molecules, which appear to play a pivotal role in iron metabolism and associated diseases. In this context, further information on physicochemical and other properties; the structure/activity correlation on the role of dietary chelating molecules such as ascorbic acid and polyphenols; new chelating drugs in addition to L1, DF and DFRA; and also metal–chelator complexes in addition to the ferric iron tri-maltol complex could be useful in the design of new therapeutic strategies for personalised medicine and overall improvement in the treatment of diseases related to iron metabolism [283,284,285,286]. Similarly, more investigations are also needed in relation to the many biological and physiological implications from the interactions of chelating molecules with iron and other endogenous and exogenous metal ions, including essential, diagnostic and theranostic metals, which influence human health and associated therapies for many iron metabolic and other diseases [287,288,289,290,291,292,293].

The interactions and effects of endogenous and exogenous chelators, as well as other metal ions, are also of crucial importance to other drugs with iron-chelating capacity such as ciclopirox, dexrazoxane and EDTA, which are widely used for other clinical conditions (Table 2). In particular, the chelator pro-drug dexrazoxane and the alpha ketohydroxypyridine chelator ciclopirox are widely used in iron-related doxorubicin toxicity in cancer patients and as an antifungal agent, respectively [294,295,296,297,298,299,300]. Furthermore, EDTA has been used for more than 50 years and is still being used in alternative medicine clinics for millions of patients worldwide (Figure 2) [301]. Changes in iron metabolism and therapeutic activity are also observed by many other drugs with iron-chelating capacity including tetracyclines, anthracyclines and hydroxyurea and also by metabolites of drugs with iron-chelating capacity such as aspirin (Table 2) [302,303,304,305].

New therapeutic strategies based on the interactions of iron-chelating drugs and other chelators with different drugs involved in erythropoiesis and other pathways of iron metabolism such as thalidomate [306,307,308], luspatercept [309,310,311,312] and hydroxyurea could increase the prospects for the design of improved therapies of affected RBC-transfused patients with refractory anaemia and iron overload. Similarly, further studies of the interactions of chelating drugs and other chelators on the activity of regulatory proteins of iron metabolism such as erythropoietin, hepcidin and ferroportin could also provide further information for the design of optimal personalised therapies in iron overload, iron deficiency and other diseases of iron metabolism [313,314,315].

In the meantime, many clinical trials in different categories of neurodegenerative diseases, kidney disease, HIV, cancer and other conditions are also in progress using mainly L1 [271,272,273,274,275,276,277,278,279,280,281,282,283]. The wide clinical interest suggests that several other diseases related to abnormal iron metabolism and iron toxicity, in addition to IDA and transfusional iron overload, can be modulated or treated using iron-chelating drugs and chelator–iron complexes. The clinical interest has recently been extended to all diseases associated with ferroptosis, which constitutes a therapeutic target for many natural and synthetic iron-chelating drugs and also chelator–iron complexes [56,57,58,59,60,61,62,63,64,65,66,67,68,69,70,71,72,73,74,75,76,77,78,79,80,81,82,83,84,85,86,87]. In this context, new therapeutic strategies in a wider range of diseases could be designed based on the progress of in vitro, in vivo and clinical trials using chelators and their iron complexes.

New recent developments affecting almost all pharmacological strategies are in progress involving the design of new therapeutic protocols, including those of chelating drugs and their iron complexes, based on precision personalised medicine and optimal therapy approaches [232,316,317,318,319,320,321,322]. These methods take into consideration clinical, immunological, microbiological, pharmacological and other parameters including ADMET drug and underlying disease characteristics [323]. Information from such data could ideally lead to the specific targeting of, for example, diseases and abnormalities of iron metabolism with different chelating drugs and also other drugs or phytochelators, as well as drug combination therapeutic strategies, which are based on appropriate algorithms and artificial intelligence techniques’ guidance for optimal therapeutic outcomes in each patient [319,320,321,322,323].

## 7. Conclusions

Recent developments in biology and medicine in relation to iron, including the discovery of new transcription factors and regulatory molecules such as hepcidin and ferroportin, and also metabolic pathways such as ferroptotic cell death, have increased our understanding of iron metabolism and its role in modulating many diseases. Similarly, several other developments in medicine in relation to iron, including the introduction of oral chelating drugs such as L1 and DFRA and also new oral chelator–iron complexes such as the ferric iron tri-maltol complex, as well as new iron diagnostic methods such as MRI T2*, have resulted in the introduction of improved personalised therapeutic protocols. All these recent developments have increased the quality of life and decreased the overall morbidity and mortality of millions of iron-loaded and iron-deficient patients. In particular, new therapeutic strategies involving effective chelation protocols of L1 and L1/DF led to the normalisation of the iron store levels in regularly RBC-transfused TM patients, which was previously considered an unattainable target.

The interest in the use of chelating drugs and especially L1 in clinical trials in patients with different categories of neurodegenerative diseases, kidney disease, HIV, cancer and other conditions with no effective therapies highlights the safety of L1 and potential application of chelation therapy in non-iron-loaded disease categories. Further studies are required regarding the interactions of iron and chelating drugs and other endogenous or exogenous chelators with regulatory proteins, dietary molecules and drugs for optimising related therapies and for designing new therapeutic strategies for ferroptosis-related diseases and other diseases of iron metabolism and toxicity.

New findings and developments, including the future introduction of artificial intelligence techniques’ guidance, will increase further the prospects for optimal therapeutic outcomes in personalised medicine using chelating drugs and other chelators in iron metabolic and many other diseases. In particular, optimal therapeutic protocols could be designed for the treatment of iron overload, IDA and other diseases related to iron metabolism, based on effective, non-toxic combinations of drugs and/or drugs with natural products, taking into consideration optimal personalised medicine characteristics such as ADMET, multi-omic and other parameters, and also many other associated variation and limitation parameters related to each disease’s characteristics.

## Figures and Tables

**Table 1:** Examples of iron complexes in medicine.

**Specific ferric iron–chelator complex drugs** Ferric iron tri-maltol. Ferric iron bis-glycinate chelate.
**Ferric iron complex drug formulations** Ferric fumarate, ferric polymaltose, iron dextran, ferric iron sucrose, ferric gluconate, ferric saccharate.
**Ferric iron complex intravenous drug formulations** Ferric iron sucrose, ferric gluconate, ferric carboxymaltose, iron isomaltoside-1000, ferumoxytol, iron dextran (low-molecular-weight forms).
**Ferrous iron complex drug formulations** Ferrous sulphate, ferrous ascorbate, ferrous fumarate, ferrous gluconate, ferroglycine sulphate.
**Dietary iron complexes** Haem (mostly in meat and blood food products). Non-haem iron complexes (mostly in vegetarian food products). Inorganic and organic iron complexes as food supplements and in food fortification.
**Iron-containing proteins (iron complex prosthetic group composition—function)** Haemoglobin (haem—oxygen transport). Myoglobin (haem—oxygen storage). Cytochromes (haem—electron transport; respiration). Cytochrome P450 (haem—drug detoxification). Ribonucleotide reductase (amino acid–iron complex—DNA synthesis). Proline hydroxylase (amino acid–iron complex—collagen synthesis). Phenylalanine hydroxylase (amino acid–iron complex—Tyrosine synthesis, deficit associated with phenylketonuria). Tryptophan 2,3-dioxygenage (haem—degradation of tryptophan). Homogentisic acid 2,3-dioxygenase (amino acid–iron complex—degradation of homogentisate in the tyrosine catabolic pathway, deficit associated with alkaptonuria). Peroxidases (haem—decomposition of hydroperoxides and use of peroxides for oxidation of diverse products, e.g., glutathione, iodide and other biomolecules). Catalase (haem—decomposition of hydrogen peroxide). Lipoxygenase (amino acid–iron complex—HPETE and leukotriene synthesis). Cyclooxygenase (haem and amino acid–iron complex—Prostaglandin and thromboxane synthesis). Adrenodoxin (iron–sulphur complex—electron transport; oxidation/reduction). Aconitase (iron–sulphur complex—Tricarboxylic acid cycle). Succinate dehydrogenase (iron–sulphur complex—Tricarboxylic acid cycle). NADH dehydrogenase (iron–sulphur complex—electron transport; respiration). Xanthine oxidase (iron–sulphur complex—conversion of xanthine to uric acid). Aldehyde oxidase (iron–sulphur complex—metabolism of aldehydes). Transferrin (amino acid–iron complex—iron transport in plasma). Lactoferrin (amino acids–iron complex—iron binding in milk and other secretions). Ferritin (Oxohydroxide, phosphate iron complex—iron storage). Haemosiderin (Oxohydroxide, phosphate iron complex—iron storage).

Note: Further information is available in references [11,12,13,14,15,20,92,97,123,125,150].

**Table 2:** Iron chelators in medicine.

**Iron-chelating drugs for the treatment of iron overload** Deferiprone, deferoxamine, deferasirox [11,149,150,151,152,153].
**Drugs with iron-chelation capacity used in other diseases** Hydroxyurea (anticancer), doxorubicin (anticancer), tetracyclines (antibacterial), ciclopirox (antifungal), EDTA (alternative medicine), DTPA (radioactive metal decontamination) [294,295,296,297,298,299,300,301,302,303,304,305,306].
**Iron-chelating pro-drugs used in other diseases** Dexrazoxane (doxorubicin and other anthracycline toxicity), aspirin (anti-inflammatory) [302,303,304,305].
**Iron-chelating proteins** Transferrin (blood), lactoferrin (milk and other secretions; neutrophils) [11,91,92,93,94,95,150,151,152,153].
**Examples of endogenous low-molecular-weight chelators** Phosphates: Pyridoxal phosphate, thiamine pyrophosphate, ribonucleoside and deoxyribonucleoside phosphates, phytic acid (IP6), pyrophosphate, ATP, ADP, AMP, etc. Amino acids: Aspartic acid, glutamic acid, histidine, cysteine, tyrosine, etc. Carboxylic acids: Citric acid, aconitic acid, oxaloacetic acid, etc. Mono- and disaccharides: Fructose, glucose, lactose, etc. Fatty acids and phosphoglycerides. Other naturally occurring chelators: Catecholamines, pteridines, purines, spermine, spermidine, glutathione, folic acid, etc. [11,20,96].
**Examples of exogenous dietary low-molecular-weight chelators** Polyphenols and other phytochelators: Gallic acid, caffeic acid, quercetin, ellagic acid, curcumin, catechin, maltol, ascorbic acid, etc. [90,176,177,178,179,180].
**Vitamins** Ascorbic acid, lipoic acid, riboflavin [168,169,170,171,172,173,174,175].
**Examples of microbial siderophores** Enterobacactin, mycobactin, aspergillic acid, etc. [149,150,181,182,183].

## Data Availability

Not applicable.

## Abbreviations

ADMET	absorption, distribution, metabolism, excretion and toxicity
DF	deferoxamine
DFRA	deferasirox
DMT1	divalent metal transported protein
DTPA	diethylenetriaminepentaacetic acid
EDTA	ethylenediaminetetraacetic acid
FR	free radical
ICOC	international committee on chelation
IDA	iron deficiency anaemia
IP6	phytic acid
L1	deferiprone
LMWt	low molecular weight
MRI	magnetic resonance imaging
RBC	red blood cell
ROS	reactive oxygen species
sc	subcutaneous
T2*	(magnetic resonance imaging) relaxation time
TI	thalassemia intermedia
TM	beta thalassemia major
